# Teenage Pregnancies in Nepal – The Problem Status and Socio-Legal Concerns

**Published:** 2018-06-30

**Authors:** Samata Nepal, Alok Atreya, Tanuj Kanchhan

**Affiliations:** 1 Department of Forensic Medicine, Devdaha Medical College, Rupandehi, Nepal

**Keywords:** *Nepal*, *patriarchal society*, *pregnancy*, *teenagers*

## Abstract

**Introduction:**

Teenage pregnancy is an issue that needs to be addressed for a better health of the women and the society. The present analysis is undertaken to find out the incidence of teenage mothers who have had hospital delivery and focuses upon the various reasons for teenage pregnancy with a brief discussion upon the associated medicolegal and social aspects.

**Methods:**

A cross sectional study was carried out in a tertiary hospital in western region of Nepal. The delivery case register were reviewed for teenage pregnancies and the relevant data was captured in a data sheet and analysed.

**Results:**

During the study period, a total of sixty-nine teenage pregnancies culminated into delivery/ childbirth. The mean age of teenage mother was 18.16+0.99 years. Majority of the teenage mothers had not completed their secondary education and were of low socioeconomic strata. Mode of delivery was caesarean section in thirty four cases, whereas vaginal delivery was conducted in thirty five cases. Live births accounted for 67 deliveries, a still birth case was of anencephalic foetus while the other one was a preterm which was spontaneously delivered at the 23rd weeks of gestation.

**Conclusions:**

Education and awareness in the form of campaign, advertisements, road shows, television or radio programmes are suggested for a decline in the rate of teenage marriages and teenage pregnancies in Nepal in the days to come.

## INTRODUCTION

Teenage mothers are those who deliver a baby before reaching 20 years of age. World Health Organization (WHO) estimated average global birth rate among 1519 year old to be 49 per 1000 girls in 2014.^1^ In Nepal, 43% of the teenage girls get married between 15 and 19 years. As per Nepalese Law, the legal age to marry for girls is eighteen years with parental consent and twenty years without their consent.^2^

Illiteracy and poverty is considered to be a key factor for early marriage in Nepal which increases the likelihood of adolescent pregnancies.^2,3^ Strong family pressure to beget a son within a year of marriage in a society where early pregnancy is perceived to have successful outcome, leaves the female under undue stress.^3,4^

Various studies have been done to see adverse outcomes and complications related to teenage pregnancies.^5,8^ The present analysis focuses upon the various reasons for teenage pregnancy with a brief discussion upon the associated medicolegal and social aspects.

## METHODS

Ethics Committee approval was obtained from the Institutional Ethics and Research Committee prior to the commencement of this study. Following the approval, a cross sectional record based study was carried out in a tertiary hospital in western region of Nepal. A review of the delivery case register was carried out daily in the Department of Obstetrics over a period of six months from July 2017 to December 2017 to identify teenage mothers who delivered during the period. The patient records/information was kept anonymized prior to analysis.

All the teenage mothers who delivered in the hospital during the period were included in the study. The present study included only those adolescent mothers who were less than 20 years at the time of delivery irrespective of their marital status and parity. The present study did not include those adolescent mothers in postpartum period, who did not deliver in the hospital where this study took place but were seeking care for postpartum or neonatal complications.

The authors did not interview the new mothers who had just undergone a stressful event of child birth. Morally, the authors feared that any personal query at that point of time will only cause psychological embarrassment to them and ethically it was inappropriate to interview the teenage mothers less than eighteen years of age. The study centre lacks electronic database system of patient treatment records/ Electronic Medical Records. All the information was thus obtained from manual search of the case files after taking permission from competent authority. The variables included in the study were age of teenage mother at delivery, marital status and literacy status, mode of delivery, gestational age at delivery, outcome of delivery and birth weight of the new born. Details lacking in the patient record regarding socioeconomic status, educational status and marital status was obtained from patient's caregivers after informed verbal consent. Modified Kuppuswamy's socioeconomic status scale was used for determining socioeconomic status.^9^ The information thus obtained was captured in a data sheet and analysed using SPSS version 21.0. The results obtained were expressed in proportions. Chi square (x^2^) test was applied to determine significance of results at P<0.05 wherever applicable.

## RESULTS

**Figure 1.**
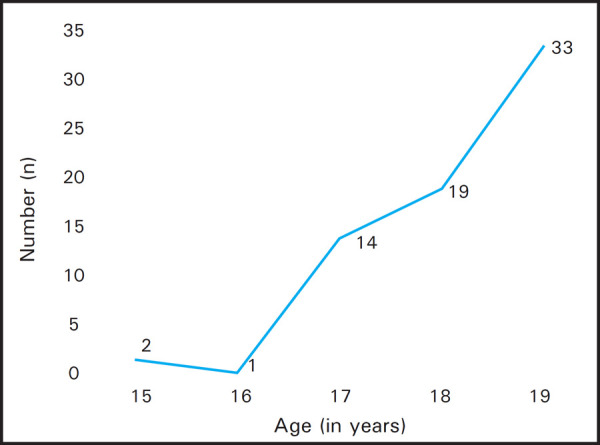
Age distribution of the teenage mothers. (n = 69)

During the study period, a total of sixty-nine teenage pregnancies culminated into delivery/ child birth which amounts to 69 (12.7%) of the total deliveries (n = 545). The majority of the cases were primigravidae (n = 66). Of the three multi gravid mothers, two had history of previous live births; a 17 year old teenager who had a female child delivered one and half years ago and the other 19 years old teenager too had a female child of 4 years at home. The third female had undergone abortion prior to this delivery. Of all the deliveries, one female had delivered twins whereas remaining were singleton deliveries. The minimum age of teenage mother was found to be 15 years with a mean age of 18.16±0.99 years (Fig. 1).

The literacy status of the pregnant teenagers was not mentioned in the patient profile. It was however noted that 17 (24.6%) teenage mothers had put their thumb impressions on the hospital consent form or birth certificate of the new-born while the others 52 (75.4%) had put their signatures on the documents. Most teenage mothers were school dropouts who had not completed their 10th standard and belonged to lower middle class of socioeconomic status (Table 1).

Most of the teenage mothers (n=64) were married. Of the remaining five females who were unmarried, one was single mother, 4 had ran away with their lovers and were living together with in-laws (n = 3), and another one was living together with her boyfriend who was from a different caste (n=1).

The various details of teenage pregnancy outcomes are depicted (Table 2).

**Table 1.** Education and Socioeconomic status.

Variable	Description	n (%)
Education	Illiterate	17 (24.64%)
	up to 5^th^ standard	11 (15.94%)
	5^th^ to 10^th^ standard	26 (37.68%)
	completed 10^th^ standard	15 (21.74%)
Socioeconomic status	upper class	2 (2.90%)
	upper middle class	4 (5.80%)
	lower middle class	31 (44.93%)
	upper lower class	18 (26.09%)
	lower class	14 (20.29%)

**Table 2.** Details of teenage pregnancy outcomes.

	n (%)
Mode of delivery	
Vaginal	35 (50.7%)
Caesarean	34 (49.3%)
Outcome	
Live birth	67 (97.1%)
Still birth	01 (01.4%)
Preterm delivery	01 (01.4%)
Gender of new-born*	
Male	40 (57.1%)
Female	30 (42.9%)

* *Twins delivered in 1 case*

Mode of delivery was caesarean section in thirty four cases, whereas vaginal delivery was conducted in thirty five cases. Out of the thirty five cases of vaginal delivery only one case was assisted with vacuum suction. Live births accounted for 67 deliveries, a still birth case was of anencephalic foetus while the other one was a preterm which was spontaneously delivered at the 23rd weeks of gestation. Birth weight recorded for the 67 new-borns from 67 deliveries including twins ranged between 950 grams and 3.7 kilograms with a mean live birth weight of 2.69±0.61 kilograms (excluding the spontaneously aborted foetus weighing 500 gm). There were 16 such neonates whose birth weight was below 2.50 kilograms, all of whom were treated with intensive care.

## DISCUSSION

Teenage girls are vulnerable population with risk for abuse, disease and desolation.^10^ They are unguarded by civil liberty and are exposed to potential harm. They are economically impoverish and emotionally vulnerable. When parents arrange marriage of their teenage daughter, there is nothing the girl could do. Higher teenage pregnancy rates in developed country is seen in economically disadvantage families, dysfunctional families and among those teenagers who are not doing well in school. ^11^ The present study observed most of the teenage mothers had not completed their secondary education. The traditional practice of dowry during marriage furthermore compels the parents to get their daughter marry early so as to save the extra expenditure on her education and food.^4,12^

Society designates an unmarried teenage girl as minor and immature, but after she is married she gets the rights and privileges of an adult. Besides, illiteracy, there are social obstacles that deter adolescent girls from seeking medical contraception.^13^ It is stigmatizing for a female to carry a condom or contraceptives. Moreover, if the female requests her partner for protective sex, she may be blamed for infidelity.^14^ Desire for virgin brides is also cited as a reason for teenage marriages and subsequent pregnancies.^15^ Societal tradition and legal practice are contradictory. A girl less than eighteen years is legally barred from having a driving licence as she is considered minor, but she is not restricted to become pregnant in her family where she is treated as adult. In this regard, it can be attributed that pregnancy in teenagers are mostly unplanned and forced rather than choice. The teenagers in the present study may be minors as per existing law, yet they are responsible adult mothers capable of making decision for their newborns.

The society in which we live has a great psychological impact upon our lives. Being pregnant attracts welcome, care and attention from husband and in-laws. The young mother may also believe being a father will make a good and honest husband. Fathering a child stirs powerful emotions of love and care in husband and psychologically he might be elated of his masculinity among his peers. These ecstatic feelings might encourage the peer group of both husband and wife for pregnancy and subsequently teenage delivery.

Teenage pregnancy has health consequences not only to the mother but also to her child.^5,16,17^ Babies born to teenage mothers are at risk of being low weight at birth and are also at risk of infant mortality.^18^ It has been observed that teenage marriage and pregnancies occur in low socioeconomic family, adolescent pregnant women neither get extra diet during pregnancy nor do they receive additional care. Being relieved from the household chores during pregnancy is also rare.

Our society and culture designates pregnancy as morally accepted practice of married people. Five teenage mothers in the present study were unmarried. Numerically, the finding of this study might be negligible but physical and psychological consequences of pregnancies in unmarried adolescents can have much graver impact on the health of the female. Interim desirability of having a boyfriend and having a baby may be another reason for teenage pregnancies. The other social issue in this regard is marriage within a same caste group. When a teenager falls in love with a person of different cast it raises serious family issues. The young lovers either have to flee from the family or forget the love of their life to marry the person chosen by their parents.^19^ The curiosity and risk taking behaviour during testosterone years is another reason for unplanned pregnancy which is also attributable to alcohol, drug abuse and/or unplanned sex.^20^

Teenage marriages and pregnancies are also a risk factor of domestic violence.^21,22^ Low intelligence and academic achievements, impulsive behaviour, physical aggression, incoherence in family and low economic status is all linked with domestic abuse. Husband may batter his teenage wife suspecting of her infidelity.

Pregnancy outside marriage is not socially acceptable in Nepal, and these girls are often treated as a social outcast. Due to the fear of being abused by parents for the taboo or the social stigma the pregnant adolescent may choose to end their life in desperation or run away from their homes. Being pregnant and delivering a baby also curtails the education lowering the educational attainment. Low level of education has its direct links to poor pay and strenuous employment, subsidized housing, poor childcare and lack of support. Having been run away from home may lead to a period of homelessness or temporary settlement at relatives. Few girls; whose family arrange a hasty marriage; survive this social catastrophe yet they are left with no choice but to abort else she will not have the family support, which in itself can be a serious concern for the health of the teenager.

Daughters born to teenage mothers have a higher risk of becoming pregnant during teenage as the young girl might be following her own mother.^6^

Discrimination based upon caste and abortions are illegal as per the Nepalese law.

Due to legal hassle and the stigma of being socially disparaged, pregnant women often choose abortions in private clinics which are unsafe when compared to general hospital. Such risky abortions are unlikely costly and go unreported. Due to poverty when the adolescent girl cannot bear the high price of illegal abortion, pregnancy is carried to full tem which increases the risk of complications related to pregnancy and unattended delivery. Babies thus born are either left in open to be abandoned by absconding mother or killed (and rarely adopted).^3^

## CONCLUSIONS

A multifaceted approach to address this complex social issue is the need of time. Illiteracy is the key issue that needs due attention in the rural society where the local residents are reluctant to discard unscientific cultural norms.

To overcome this, sex education is to be made mandatory in the curriculum of adolescents, who are to be taught the ills of childhood marriages that are not only legally punishable but also complicates the life of the teenager, mentally, physically and economically.
